# How palliative care patients’ feelings of being a burden to others can motivate a wish to die. Moral challenges in clinics and families

**DOI:** 10.1111/bioe.12590

**Published:** 2019-05-21

**Authors:** Heike Gudat, Kathrin Ohnsorge, Nina Streeck, Christoph Rehmann‐Sutter

**Affiliations:** ^1^ Hospiz im Park Hospital for Palliative Care Arlesheim Switzerland; ^2^ Institute of Biomedical Ethics and History of Medicine University of Zurich Zurich Switzerland; ^3^ Institute for History of Medicine and Science Studies Lübeck Germany

**Keywords:** burden to others, end of life, ethics of care palliative care, qualitative studies, wish to die, wish to hasten death

## Abstract

The article explores the underlying reasons for patients’ self‐perception of being a burden (SPB) in family settings, including its impact on relationships when wishes to die (WTD) are expressed. In a prospective, interview‐based study of WTD in patients with advanced cancer and non‐cancer disease (organ failure, degenerative neurological disease, and frailty) SPB was an important emerging theme. In a sub‐analysis we examined (a) the facets of SPB, (b) correlations between SPB and WTD, and (c) SPB as a relational phenomenon. We analyzed 248 interviews with 62 patients, their family caregivers, and professionals using grounded theory and interpretive phenomenological analysis. SPB appeared as important empathic concern in care situations. Patients expressed many sorts of concerns for others, but also perceived an altered self‐understanding that did not meet mutual expectations within relationships. In SPB associated with WTD three constellations were found: (a) WTD to unburden others; (b) patients decided against hastening death to prevent being a further burden to others (in these cases, the SPB counteracted the wish to die); and (c) both wishes for and against dying were sustained by SPB. These patients often felt paralyzed and suffered deeply. Family caregivers felt emotionally touched by SPB and tried to unburden patients by caring and compassion. We concluded that the impact of SPB on a WTD and the various meanings the facets of SPB have in balancing relationships need to be worked out individually. An early palliative and narrative approach is warranted.

## 1 INTRODUCTION

It is well known that the feeling of being a burden to others is frequently experienced by patients in palliative care and 19–65% of them say they experience strong negative feelings, or even suffering, from their belief that they are a burden to those who care for them.1McPherson, C. J., Wilson, K. G., & Murray, M. A. (2007). Feeling like a burden: Exploring the perspectives of patients at the end of life. *Social Science & Medicine*,* 64*(2), 417–427.


The feeling of being a burden, or self‐perceived burden (SPB), is a common phenomenon in patients with advanced and terminal illness, especially with cancer, motor neurone disease, stroke, chronic pain, and frailty.2Bausewein, C., Calanzani, N., Daveson, B. A., Simon, S. T., Ferreira, P. L., Higginson, I. J., … PRISMA. (2013). “Burden to others” as a public concern in advanced cancer: A comparative survey in seven European countries. *BMC Cancer, 13*(105). Retrieved from http://www.biomedcentral.com/1471-2407/13/105. Kowal, J., Wilson, K. G., McWilliams, L. A., Péloquin, K., & Duong, D. (2012). Self‐perceived burden in chronic pain: Relevance, prevalence, and predictors. *Pain*,* 153*(8), 1735–1741. It arises when patients have to deal with physical distress or with psychosocial and existential concerns, and correlates with anxiety, depressive symptoms, hopelessness, suicidal ideation, and caregiver burden.3Chochinov, H. M., Kristjanson, L. J., Hack, T. F., Hassard, T., McClement, S., & Harlos, M. (2007). Burden to others and the terminally ill. *Journal of Pain and Symptom Management*,* 34*(5), 463–471. Chio, A., Gauthier, A., Calvo, A., Ghiglione, P., & Mutani, R. (2005). Caregiver burden and patients’ perception of being a burden in ALS. *Neurology*,* 64*(10), 1780–1782. Kowal, *et al.,* op. cit. note 2. Ruijs, C.D.M., Kerkhof, A. J. F. M., van der Wal, G., & Onwuteaka‐Philipsen, B. D. (2012). The broad spectrum of unbearable suffering in end‐of‐life cancer studied in Dutch primary care. *BMC Palliative Care*,* 11*(12). Retrieved from https://doi.org/10.1186/1472-684x-11-12.


SPB significantly influences decision‐making in serious illness, including decisions about where to live4Murray, M. A., O'Connor, A., Fiset, V., & Viola, R. (2003). Women's decision‐making needs regarding place of care at end of life. *Journal of Palliative Care*,* 19*(3), 176–184. and whether to prolong or withhold life‐sustaining treatment.5Zweibel, N. R., & Cassel, C. K. (1989). Treatment choices at the end of life: A comparison of decisions by older patients and their physician‐selected proxies. *Gerontologist*,* 29*(5), 615–621. It has an impact on interpersonal functioning in a family system6Cousineau, N., McDowell, I., Hotz, S., & Hebert, P. (2003). Measuring chronic patients’ feelings of being a burden to their caregivers: Development and preliminary validation of a scale. *Medical Care*,* 41*(1), 110–118. McNamara, B., & Rosenwax, L. (2010). Which carers of family members at the end of life need more support from health services and why? *Social Science & Medicine*,* 70*(7), 1035–1041. and it is known as a significant predictor of suicidal ideation and death‐hastening acts.7Ganzini, L., Beer, T. M., & Brouns, M. (2006). Views on physician‐assisted suicide among family members of Oregon cancer patients. *Journal of Pain and Symptom Management*,* 32*(3), 230–236. Wilson, K. G., Curran, D. McPherson, C. J. (2005). A burden to others: A common source of distress for the terminally ill. *Cognitive Behaviour Ther*apy, *34*(2), 115–123. Wilson, K. G., McWilliams, L. A., & Péloquin, K. (2013). Chronic pain and the interpersonal theory of suicide. *Rehabilitation Psychology*,* 58*(1), 111–115. McPherson, C. J., Wilson, K. G., & Murray, M. A. (2007). Feeling like a burden to others: A systematic review focusing on the end of life. *Palliative Medicine*,* 21*(2), 115–128.


Christine McPherson has defined the feeling of being a burden to others, the self‐perceived burden (SPB), as an “empathic concern engendered from the impact on others of one's illness and care needs, resulting in guilt, distress, feelings of responsibility, and diminished sense of self.”8Op. cit. note 1. This description of the SPB has its place in care relationships: people care for each other, or more particularly, in the case of illness, in practice one person takes over the care of somebody who is ill. In SPB, at least in theory, somebody who is in need of care and receives care, cares for those who care.

In a qualitative interview study of 15 cancer patients, McPherson9Ibid. found that SPB as a relational phenomenon included the concerns for others, but also the implication for the self that covered self‐perceived responsibility towards those who cared, and that could lead to a diminished sense of self. She argues that patients develop strategies in order to maintain reciprocity within their relationships. McPherson's interesting work is one of the very few qualitative studies investigating in depth the phenomenon of SBP in patients with cancer,10Ibid. but does not focus on wishes to die (WTD) in particular. Conversely, other studies do report the importance of SPB in the etiology of WTD, but do not investigate the phenomenon in more depth.11Rodriguez‐Prat, A., Balaguer, A., Booth, A., & Monforte‐Royo, C. (2017). Understanding patients’ experiences of the wish to hasten death: An updated and expanded systematic review and meta‐ethnography. *BMJ Open*,* 7*(9), e016659.


We report here some results from an interview study12Ohnsorge, K., Gudat, H., & Rehmann‐Sutter, C. (2014). What a wish to die can mean: reasons, meanings and functions of wishes to die, reported from 30 qualitative case studies of terminally ill cancer patients in palliative care. BMC Palliative Care, 13(38). http://www.biomedcentral.com/1472-684X/13/38. that investigated the WTD aims of terminally ill patients. The aim of this study of 62 patients, their nurses, physicians, and family caregivers, was to understand what these patients think and intend when experiencing a WTD. The feeling of being a burden to others emerged as an important theme in many interviews when investigating the motivations behind a WTD, but also independently of WTD.

Here we present an in‐depth analysis of this SPB sub‐topic. We presume that a better phenomenological understanding of SPB may have practical ethical implications for several reasons: It may facilitate communication about these feelings in the complex webs of relationships. It may also help to detect the underlying moral understandings and the needs of those involved, and may therefore make better care possible for these persons and their families. In the discussion, we show that SPB is based on conflicts of values and moral understandings, including moral claims towards oneself and others. These claims often have a significant influence both on what persons at the end of their life wish and their actual decision‐making. In caring for someone with a WTD, awareness of SPB may enable a better understanding of the values and moral sensibilities motivating this wish, and allow carers to be responsive to the particular needs and experiences of the ill person and their families.

## 2 METHODS

In our qualitative, prospective, interview‐based study we investigated in detail what patients with advanced and terminal cancer and non‐cancer disease mean when they express a WTD. We combined phenomenological and hermeneutic approaches, inspired by interpretive phenomenological analysis and grounded theory. The details are described elsewhere.13Ohnsorge, K., Gudat, H., & Rehmann‐Sutter, C. (2014). Intentions in wishes to die: analysis and a typology ‐ a report of 30 qualitative case studies of terminally ill cancer patients in palliative care. Psycho‐Oncology, 23, 1021‐1026. We interviewed 62 patients and their informal and professional caregivers (248 interviews). The patients suffered from advanced cancer (n = 30), organ failure (n = 11), degenerative neurological disease (n = 10) or frailty (n = 11), lived in different settings, and all had access to palliative care support. At the start the participants were enrolled covering a broad range, and simultaneously with data interpretation. Over the course of the study they were selected increasingly narrowly according to whether they had a WTD or not. Face‐to‐face interviews were conducted by two trained interviewers based on a semi‐structured interview guide, audio‐recorded, and fully transcribed. If possible, the patients were interviewed several times; the intervals were adapted to the development of the disease and turning points in the patient's situation. Important topics could be narrated as they arose.

All the interviews were analyzed by three or four researchers using interpretative phenomenological analysis14Smith, J., Flowers, P., & Larkin, M. (Eds.), (2009). *Interpretive phenomenological analysis. Theory, method and research*. Los Angeles, CA: Sage. and grounded theory methodology,15Charmaz, K. (2006) *Constructing grounded theory. A practical guide through qualitative analysis*. London: Sage. first by case, then by case groups. We used an interpretative phenomenological analysis approach for the general analysis. Each researcher undertook an analysis of the individual case, and these different interpretations were then shared in a case discussion in the research team. From these discussions, memos containing the shared interpretation were written for each individual case. Emerging themes were developed. The triangulation of interviews from patients, relatives, and professional carers provided additional information and a richer view of the case. Interviews with relatives and professionals were taken as independent perspectives, but also gave insight into interpersonal communication, caregivers’ experience, relational issues, and interactions on the perspective of the patient. In order to compare the data between the different patient groups we then applied an analytic tool developed in the first part of the study. The development of this model from the data was based on a constructivist approach to grounded theory (see our publications elsewhere16Op. cit. note 12. ).

“Feeling like a burden to others” was one of the most important emerging themes. It arose when we analyzed the data in terms of the research sub‐question about factors that triggered or prevented the patient's WTD. We then decided to investigate the theme of SPB separately and undertook a refined analysis of the material with regard to (a) the patients’ experience of SPB, independent of the existence of a WTD, (b) SPB in relation to a WTD, and (c) the relational aspects of SPB. Based on this analysis, we constructed sub‐categories for the entire theme of SPB, which we report here in the results section. The interviews did not ask proactively whether patients felt they were burdening others, but did investigate this further if patients spontaneously mentioned it. Their accounts gave insight into the relational and decisional context and the moral understanding and expectations behind feelings of being a burden.

With regard to SPB, the interviews with the caregivers were used to complete the patients’ view of the situation but also to illuminate their own perspectives. In one of the main questions, caregivers were asked whether they talked with the patient about their WTD, and how they coped with the whole situation. If feelings of being a burden were mentioned by the caregiver we investigated further, through questions that probed what the caregivers had reported. In these cases, for example, we asked whether and how caregivers perceived the patients’ attitude about SPB, how burdensome caring actually was for them, and the impact of the relational interplay.

## 3 RESULTS

In total, 31 out of 62 patients mentioned a self‐perceived burden (SPB). In four further cases, the patients did not disclose their SPB clearly, but the family members who cared for them reported it in the interview; everyone added that this affected them deeply. Of the 31 patients with SPB 18 also expressed a WTD in the present or in a future situation that seemed already very concrete to them. The other 13 patients with SPB described other wishes: a wish to live, acceptance that death would come without wishing it, or other constellations that could not be adequately clarified. Apart from the 31 cases with SPB, 12 patients mentioned a WTD without any feelings of being a burden to others.

Although patients described in direct words how burdened they were by symptoms or impaired activity, they rarely explicitly said, “I am a burden”, or “I burden others.” Feelings of being a burden to others were conveyed through story‐like accounts about events and experiences. They stood in a broader psychosocial and spiritual context, including living conditions, cognitive functioning, preexisting couple conflicts, disease trajectories, gender issues, and culture.

The in‐depth analysis of the interview quotes about feeling like a burden revealed four sub‐topics, which we expand below:

- Facets of self‐perceived burden: What do people experience when they feel SPB?
- The correlation between SPB and WTD in advanced illness
- The relational dimension: interaction of patients and family members in the context of patients’ perceived burden
- Strategies of patients and relatives to counter SPB

### 3.1 Facets of SPB

Growing illness‐related fragility and the complexity of daily life influenced the patients’ relationships with their family, friends, and the broader environment. New balances within these relationships had continually to be found. However, the patients perceived their own space of agency to contribute to a balanced relationship as increasingly limited. They addressed two main concerns when they spoke of SPB. First, they were seriously concerned that they would burden others by causing hardship in many different ways for whoever cared for them. Second, we found persons speaking about SPB when they could no longer hold a positive, meaningful image of themselves within the relationship—instead, they perceived themselves to be solely the cause of problems and disappointment. In the clinical routine, an archetypal phrase for the first theme would be, “I worry that my wife is so exhausted by the care she has to give me”; for the second it could be, “I see no sense in life. I have nothing useful to do. They don't need me. I always took care of everybody, now they have to do everything for me.”

#### 3.1.1 Concerns for others

Many patients described their SPB as being motivated by their serious concern for others whom they knew were affected, sometimes severely, by their illness and/ or by caring for them. They believed others to be burdened in physical, emotional, spiritual, social or other ways.

*Causing a physical burden*. Ill or frail persons with SPB were concerned about the physical exhaustion of their family caregivers, even if they were well supported by professionals. Patients worried that others might suffer from heavy physical labor, undertaking the technical support of tubes or machines (respirators), transport, or fatigue due to sleep deprivation by providing night‐time assistance.

*Causing an emotional burden*. Patients perceived grief, fear of loss, spiritual pain, and helplessness among the family caregivers, but also guilt, anger and disappointment. Especially when connected with a WTD, statements about being a burden to others could trigger separation anxiety, grief, and emotional instability on the part of the family member. This in turn could provoke feelings of guilt and withdrawal by the patient. For many patients and caregivers, it was difficult to talk about the emotional burden. This led to more indirect communication, mutually unclarified assumptions, relational misunderstandings, speechlessness on both sides, and withdrawal, as the example of this 66‐year‐old woman (P8‐II) with motor neurone disease shows. She described how her husband consistently avoided speaking about her illness because it would break his heart:

He's in a panic at losing me, and he's protecting himself by not wanting to know about anything. Well, I mean, how I am, but not how things will go on. […] That's terrible for me, because I can't talk about it with him. I can't [talk] about the fears that come up sometimes, of not being able to talk any more, of not being able to do this, or do that. So, I'm not allowed to say anything to him, because he can't cope with it.

*Being a social burden*. Patients developed feelings that they were burdening their family caregivers because traditional roles had changed, or they assumed they had created too much or “unsuitable” work. This often also implied gender aspects, as shown by this quote by a 57‐year‐old woman with cancer (P18‐I), who said of her caregiving husband:

It might be a lot if you [the husband] have to work all day, and then you come home and have to cook and do this and that.

The ill persons reported that it was difficult for relatives if too many professionals disrupted their privacy at home, and they therefore sometimes went so far as to reject external help. This resulted in difficulties in finding the right mix between important interests: preserving privacy for family members while avoiding overloading them, but also realizing that professional care preserves a patient's own integrity and self‐control.

*Being a burden because of a future that cannot be controlled*. Some patients feared an increase in further care needs and dependence as the illness progressed, or were concerned for their loved one's future financial shortcomings, prolonged mourning or psychological problems due to their own death, especially for patients with young children.

#### 3.1.2 Conflict with one's self‐understanding

Some patients felt they were a burden when their own self‐worth was so weakened that they no longer perceived their own self‐image within their relationship to be positive. Bodily and cognitive decline, loss of spiritual orientation, social deprivation, financial difficulties and self‐blame about self‐inflicted disease (for instance having caused cancer by smoking or failing to have medical check‐ups) discouraged the patients from feeling like a valuable partner in their relationships. A frail elderly woman (P33‐II) expressed her guilt at burdening others with her need for round‐the‐clock care:

For 2 weeks, I had to keep disturbing them at night even though I didn't want to. My daughter‐in‐law has a household too, and she goes to work, and it became very uncomfortable for me. […] Heck, if she has to come and clean up three times [because of the incontinence]. […] You feel guilty, even if you know you can't do anything about it.

Other patients felt they had failed in what they perceived to be their social responsibility, and that they were burdening others with a loss that was too early and too heavy. Still others said that just imagining that they provoked disgust during intimate care from the smell of a stoma or wound, a distorted body or behavioral changes, made them feel guilty, ashamed or helpless, and they concluded that they were a burden. One example was this 76‐year‐old woman (P4‐I), who admitted that shame about her ulcerated and foul‐smelling breast cancer was a reason for her WTD:

Yes, well, I've thought umpteen times that I'd like to have someone from Exit [a right‐to‐die organization] come […] because I thought: Yes, well, if it's so unbearable that, that everyone around me has to hold their nose. That was the worst, I think, then I just wanted to, break off, the exercise. […] I have the feeling that you also have the right, if you feel, I'm so burdened myself that I burden other people like this, I'd like to end this now.

### 3.2 Correlations between SPB and WTD

More than half of the patients who expressed SPB developed a WTD. In most cases, as well as SPB, there coexisted other important conditions, such as a high symptom burden, care dependency, immobilization, and spiritual or existential concerns that were interconnected and influenced each other.

We found three different constellations between SPB and concerns for others: (a) some patients expressed a WTD motivated by the feeling of being a burden to others; (b) in other cases, patients expressed WTD for other reasons, but refrained from putting their WTD into action because they perceived that doing so would burden their caregivers (in these cases, the feeling of being a burden was not a reason for a wish to die, but counteracted it); and (c) Some very burdened patients remained in a paradoxical situation. Their WTD was motivated by SPB, but at the same time they decided to keep on living, because hastening death would be too much of a burden to the relatives. Both wishes (for and against dying) were sustained by SPB, and patients described deep feelings of suffering due to this.

#### 3.2.1 Burden to others as a reason to wish to die

Some patients wished to die or even to hasten death primarily to reduce the burden on the family, their professional caregivers, or society. An elderly woman with a brain tumor (P2‐I), who had always defined herself as somebody who was there for others, had difficulty coping with her new dependency, and therefore wished to die:

I always pray that I can release people, eh, that I can free them from a burden, release the others as well. So that I don't always have to rely on help, I want […] My whole life, I only worked and always took care [of others] myself. […] Then after this it's just difficult, if you always have to have other people. Having to be a burden. […] That I've never liked.

A male patient aged 87 with lung cancer (P22‐I), living alone, planned assisted suicide after a crisis at home, but was convinced by his general practitioner to be hospitalized first in a hospice. Even though the WTD diminished under hospice care, during each morning round he said he felt he was a burden to society as a sick person in need of care, and also as an elderly person who was no longer productive but just causing costs to society. He explained:

Death is a terrible thing, everything is destroyed, it's like a library that's burned and only the ashes remain. […] I could imagine a spiritual world where you can just keep living, reading thoughts without having to be a burden to others with high costs of aging.

This patient case is reported elsewhere in detail.17Gudat, H. (2015). From understanding to patient‐centered management: clinical pictures of a wish to die. In Rehmann‐Sutter, C., Gudat, H. & Ohnsorge K. (Eds.), The Patient's Wish to Die. Research, Ethics, and Palliative Care (1st Ed.). Oxford: Oxford University Press.


#### 3.2.2 Reasoning against a wish to die

Among the persons we interviewed, however, it was more common to find that the SPB counteracted a WTD or even to hasten death. Some participants worried that their WTD or hasten death would give others additional burdens. Because of these worries, they decided not to act on their wish to hasten death. A 65‐year‐old woman (P17‐I) with pancreatic cancer, for example, had an intense wish for death to come soon. She grounded her reasoning against death‐hastening activities in her experience of a suicide attempt years before:

No, I knew that if I jumped from our building, that would be terrible for all those who live around, who knew me. No! And for those left behind that is quite terrible! I would not want to hurt anyone. I tried that once and everyone suffered a lot. No, I certainly would not do that again. […] Dignity was always important for me throughout life, and I was ashamed for years afterwards.

Other patients avoided expressing a WTD and suffered, because their family had signaled that death‐hastening ideas or actions would make them feel they had failed, or because the family associated a good death with a natural death.

Still other patients were able to relativize their wish to die, because the love of family members and of obligations toward them prevailed in positive and unburdening ways. A 67‐year‐old widower (P5‐II), severely handicapped by advanced multiple sclerosis and with a profound wish to die, said:

It [the WTD] is always there – more or less. There are moments when I'm feeling better, for example, when I can go to my daughter, then I certainly don't have these thoughts. […] It's so nice with my daughter and her boyfriend, that it [the WTD] only comes back [later].

#### 3.2.3 Burdened by wishes for and against dying

Some patients were heavily burdened by their own situation, felt it would burden others, and therefore wished to put an end to an unbearable situation. At the same time, they realized that their WTD in itself was heavily burdening their loved ones. These patients felt hopeless and unable to act; they suffered deeply. A young cancer patient (P19‐I) explained her wish to be dead as because of her unmanageable symptoms, loss of self‐control, and strong feelings of guilt in having failed as a caring mother, daughter, and spouse:

I was just thinking whether it would be easier for everyone if I was not there anymore and everyone could concretely plan their life further. My mother would simply need to accept the fact [of my death], my husband and my child could stop the hospital visits and could approach the path from which we do know that it will come.

But at the same time, she knew that actually hastening death herself would burden her family even more:

It is already difficult enough for the child, having to know later on that his mum died, but then also to know that she did it herself, prematurely, when I could have shared a few days more with him […] My husband would think: if only I could have cheered her up more, then she might not have left. I think that [hastening death] adds to the grief of relatives even more, *[pause]* many thoughts that are unnecessary.

Regarding a connection between SPB and WTD, we observed differences between groups enduring different trajectories of illness.18Ohnsorge, K., Rehmann‐Sutter, C., Streeck, N., & Gudat, H. (2019). Wishes to die at the end of life and subjective experience of four different typical dying trajectories. A qualitative interview study. PLoS ONE 14(1), e0210784. https://doi.org/10.1371/journal.pone.0210784. While only one out of 11 patients with organ failure complained of SPB and also expressed a WTD (a further five patients with SPB, but without a WTD), we found this combination in six of 10 patients with neurological diseases. Patients with cancer and frail elderly people were in the middle range (cancer 9/30 patients, frail elderly 3/11).

### 3.3 To feel like a burden and to be burdened as a relational phenomenon

Care situations in advanced illness substantially change the equilibrium of give and take in a relationship. Partners have to find and accept a new equilibrium that is meaningful to them, despite its disparities.

Family caregivers tried using all means to avoid generating feelings of being a burden, and if patients mentioned such feelings, caregivers would say that they were unfounded. One could assume that the load of care was not the crucial issue for the caregiver, but that the burden came from the fact that the patient's self‐worth (e.g., through incontinence or loss of control) or basic values (e.g. being autonomous or fulfilling an active role in the family or in social life) were compromised and that family caregivers felt unable to prevent this. Family caregivers felt concerned, but often speechless and helpless. The spouse of a patient with motor neurone disease described such an episode (P9‐II):

I didn't think it [wetting himself] was great at first either. But afterwards I said: “It's over, it's fine”. *[pause]* Then he cried and said: “Yes, it's time for me to go soon.” Then I said: “Don't ever say that again! That isn't a reason!”

We found family systems in which the partners had found new, meaningful ways to relate to one another, despite heavy symptom load and the need for care. These patients and caregivers expressed the positive meaning the relationship had for them, strengthened by a common vision. In other relationships, there was discord between partners, for example about their definition of reciprocity within the relationship, or the perceptions of mutual needs. In these situations, everyone concerned seemed more burdened, and WTD caused additional stress to the caregivers. In such cases family caregivers were then sometimes so burdened that they refused an interview.

#### 3.3.1 SPB in relationships that were perceived as meaningful

In these constellations care efforts and symptom burden could be high, but if patients expressed their SPB they could nevertheless cope with them. These patients typically lived in a partnership and reported strong family ties; some of them emphasized how important it was to them to look back on a fulfilled life or still to have a task, if only as a recipient of love (for example, as a grandfather). Patients expressed their admiration for their caregivers’ efforts.

The family caregivers conceded that care‐giving was sometimes exhausting, but more importantly, giving care and support saved them from resignation and feelings of helplessness when confronted by their loved ones’ suffering. Caring helped them to feel in control of a difficult situation, rather than being at its mercy. They preserved an active role for themselves by managing the care, being informed about diagnosis, treatment options and prognosis, and by cherishing good moments. Despite all the grief and sorrow, some caregivers and patients experienced their final time together as spiritually fulfilling, with a shared purpose and a desire to spend the remaining time together.

#### 3.3.2 SPB in relationships that were perceived as unbalanced

In situations in which patients for some reason felt the relationship was unbalanced or perceived a strong (often unspoken) value conflict, they often reported being burdened themselves and/or had strong feelings of being a burden to others. The family caregivers of these patients all confirmed that the physical and emotional demands were high. In some cases, patients and caregivers seemed to have different agendas and purposes, communication was disrupted (sometimes through mental impairment), and unspoken assumptions led to misunderstandings. In some cases of very burdened patients or families, the family caregivers refused to give an interview because either the patients or the family members themselves regarded the interview as yet another burden.

#### 3.3.3 Speechlessness

Patients reported that the feeling of burdening others was one of the hardest topics to speak about with their family. Emotional burden on others, in particular, provoked secondary feelings of guilt, shame, anger or hopelessness. Both sides felt a speechlessness that left them with burdensome loneliness. The ill person and the caregivers then took adaptive actions, based on assumptions about the others’ needs. The caregivers, for example, intensified care to an exhausting level, while patients pretended to be content, did not call for help, and concealed their feelings of being a burden; and WTD remained unspoken in order to prevent further destabilization of the situation. A 66‐year‐old woman (P8‐II) with motor neurone disease told us:

I just have to take care that I don't say too much to my husband. Because he can't change it either, and it's bad for him too. And then he doesn't know what he's supposed to say, he's helpless as well. […] That's terrible for me, because I can't talk to him about it. I can't [talk] about the worries that come sometimes. About not being able to talk any more, about not being able to do that [physically] any more, I can't say anything about this to him, because he can't cope with it.

#### 3.3.4 Distancing

To know the heavy burden that the other carries, and to be the cause of it, was terrible for many patients. Some patients thought that the best way to resolve the hopeless situation would be to “wean ourselves off each other” (P1‐II), for example by restricted contact or even by death. One patient wrote letters to his grieving wife, signing them with “your best friend” instead of “Your X”, explaining to her that creating a distance early on would lessen the shock “when one of us dies.” A woman with motor neurone disease (P1‐II), confronted with the announcement that her partner would commit suicide if she died, tried to unburden him using the following strategy:

The problem is, he says: If you die, I don't want to live any more. That is my enormous problem. So, I can't even die in peace. […] But I know my path will slowly move away from him. And I have— consciously— separated myself from him a little, so as not to make it hard for him. But then he suffered terribly and said: “You're not the same any more.” […] And I often hurt him, deliberately, in order to create this distance. Because it's still ringing in my ears: “I'll kill myself.” And because I'm so afraid of that, I treat him so, so [*makes a gesture of throwing something away*].

### 3.4 Strategies against feelings of being a burden

Patients told us about various strategies to cope with the feeling of being a burden to others: they sought to stay active, maintain control, and put things in order. If necessary, they minimized their own needs, hid their discomfort, or avoided other people witnessing their suffering. Some patients mentioned how they tried to minimize their caregivers’ physical load as far as possible, even at the cost of their own wellbeing, such as not calling for the caregivers’ assistance when it was actually needed. It relieved them of a burden when caregivers accepted external care.

Most importantly, however, the feeling of being a burden frequently influenced important decisions by persons at the end of life: decisions regarding withholding or withdrawing life‐sustaining treatment, particular decisions in advanced directives, choosing institutionalized care, or desiring to hasten death. The woman with MND described above (P8‐II), for example, included a do not resuscitate (DNR) order in her advance directive, with the argument that it would spare herself and her partner years of suffering and caregiving:

My husband nearly freaked out [when he heard about the DNR order] and then I said, you simply have to respect that [DNR order], because that saves me a bunch of years of suffering, me and you, because he would need to take care of me and then he is indeed no longer free.

The daughter of another woman with advanced breast cancer (P4‐I) said that behind her mother's wish to hasten death was the idea that she could not imagine her husband witnessing her illness and caring for her at the same time:

I think mainly that she wanted to go easy on her husband and me, because she, well she also told me several times: I don't think that your father, I mean her husband, would be capable of nursing me, and seeing me if I'm in such a bad way, I mean keeping me at home.

## 4 DISCUSSION

The feeling of being a burden to others was common both in cancer and non‐cancer patients. Similarly, as McPherson19Op. cit. note 1. has shown, the patients in our study were concerned not only by the burden they perceived on those who cared for them; they also perceived a change in self‐understanding, such that mutual expectations (or what they understood by them) within their relationships were not met. The self‐perception of being a burden was associated with strong emotions such as guilt, worry, shame, anger, self‐hate, self‐doubt, and disappointment, but—and this is important—also with more positive feelings of loving concern and solicitude.

Frequently, however, these emotions indicated a moral conflict that the person with SPB was experiencing, in the sense that they felt that moral values important to their self‐understanding and associated moral claims, were being violated. For example, some SPB was explained by the experience of a loss of autonomy and the feeling of loss of self‐worth. Behind this stood the moral self‐claim: “I should be or want to be autonomous and independent.” Others explained they felt they were a burden to their loved ones since they could no longer fulfill their family responsibilities (such as a mother whose illness meant she could not care for her toddler), and they had a deep feeling of failure. These feelings were based on important moral values such as care, reciprocity, and responsibility towards others, especially towards the vulnerable, care‐deserving family members. It included the moral claim: “I should help my loved ones and fulfill my social roles and responsibilities towards them in care relationships.” Others, due to their illness or frailty, suffered from the loss of what they perceived as a useful social existence. This was based on the value of being able to live an active life and contribute meaningfully and productively to society. The underlying moral self‐claim was: “I should contribute meaningfully, actively and productively to society.” Still others saw not being a burden as a moral duty, which was closely linked to how they saw themselves in relation to others, with the underlying moral claim: “People should be free to live and decide for themselves without being constrained by others.” It might also have roots in claims towards themselves such as: “Don't give other people trouble.” The fact that people felt these important values and inherent moral claims had been violated generated their feeling of being a burden to others.

We conclude from this that SPB can be traced back to a person's moral reasoning. To understand SPB it may be helpful to see it as a moral conflict involving the violation of an important moral claim or conflict between important moral values and claims towards oneself and others. Identifying and making explicit these conflicting values and moral understandings might be a starting point for a dialogue about the relational and care needs behind SPB.

This insight may ultimately be helpful not only for improving communication between the persons involved, but also for exploring how to sustain the patient best in her particular moral understandings and autonomy at moments of important decision‐making. SPB— as we have been able to show— influences significant decisions of persons undergoing serious illness: for example, decisions about the withholding or withdrawal of life‐sustaining treatment, choosing advance directives or institutionalized care, or enacting a WTD.

Our results also show that besides the way that SPB reflects inner value conflicts, it is often equally influenced by tensions in relationships in which the new attribution of roles, of giving and receiving, of constricted freedom and unchosen duties may contrast with people's ideals of a good relationship at times when they were well. Whenever people could not find new ways towards meaningful relationships in times of illness, when communication broke down or people distanced themselves from each other, SPB was much more likely to be experienced.

In some settings, patients and family caregivers therefore seemed to be caught up in a spiral of burdening and being burdened, and patients often said they wanted to die in order to unburden others. This raises the question of why the patient and family did not change the setting, although help was offered. Only understanding in detail the reasoning of all those involved gave insight into the fact that the concern about burdening others is part of the complex balance of coping with severe illness and part of the coping behavior in a specific family system.

The results show that SPB is a complex phenomenon. It often consists of a cluster of emotions, thought constructs, and actions in which concerns for others play an important role. It is important to understand that feelings of being a burden reveal a conflict between important values and moral understandings of the self in a relationship in the context of love, care, and illness. These feelings may be generated or escalated via alternating assumptions of the patient and those in her surroundings (the real and perceived burden on the others, and how others react to the patient's SPB). But also other factors are relevant, such as psychological wellbeing, cognitive functioning, preexisting conflicts between couples, illness trajectories, gender issues, and sociocultural background.

SPB was frequently given by patients as a motivation for their WTD. But depending on which values were prevalent, it could also counterbalance a wish to hasten death, when concerns about causing harm to others were perceived as outweighing what one wished for oneself. Our results show that it is important to acknowledge that SPB does not necessarily lead to a WTD, but in fact often prevents the wish being acted upon. In these situations, people are weighing up different values important to their self‐understanding (importance of caring, of being loved, reciprocity, responsibility towards others versus autonomy, dignity and self‐worth). The perception of being a burden can therefore be associated with a WTD but, surprisingly, can also prevent or suppress it. Other authors have described the reassuring reverse side of SPB, stressing the relational aspect of care and love and their meaning in sense‐making at the end of life.20Akazava, T., Akechi, T., Morita, T., Miyashita, M., Sato, K., Tsuneto, S., … Furukawa T. A. (2010) Self‐perceived burden in terminally ill cancer patients: A categorization of care strategies based on bereaved family members’ perspectives. *Journal of Pain and Symptom Management*. *40*(2), 224–234. Ruijs, *et al*., op. cit. note 3.


## 5 CLINICAL ETHICAL IMPLICATIONS

In our study, about half the patients expressed SPB, some of them also mentioning a WTD (with or without concomitant wishes to live) and others having a wish to live or accepting that death would come. Ethically it is not plausible that WTDs in the context of SPB and advanced disease are always bad. They can be a way to accommodate to an inevitable ending of life.21Rehmann‐Sutter, C. (2015) End‐of‐life ethics from the perspectives of patients’ wishes. In C. Rehmann‐Sutter, H., Gudat, & K. Ohnsorge (Eds.) *The patient's wish to die. research, ethics, and palliative care* (1st edn) (pp. 161–170). Oxford: Oxford University Press. A WTD can be also seen as an invitation to talk to one another. Most patients who express a WTD do not intend to hasten death actively right at that moment, but to stay part of a process through which they negotiate an unfamiliar, unstable life situation and its approaching end. Most people feel less alone in this transitional phase if they can talk openly.

Patients assume that expressing a WTD will burden their caregivers. However, to disclose such a wish also means that there is enough trust within a relationship to talk openly. Perhaps professionals should worry instead about patients and families who are heavily burdened, and where thoughts about dying cannot be discussed because talking about it is too difficult or communication within the family is compromised. In these cases, a language needs to be found in the patient–physician relationship that respects the vulnerability of the family system, including an approach to moral beliefs, intra‐familial relationships and communication. Clinical interaction needs to create a space of healing.22Churchill, L. R., Fanning, J. B., & Schenk, D. (eds) (2013) *What patients teach. The everyday ethics of health care*. Oxford: Oxford University Press. Patients and their caring families should be supported with empathy at these moments. What grounds an SPB with and without a wish to die, and which values and moral understandings motivate it and may be in conflict, needs to be worked out for each individual. As our patients’ and caregivers’ interviews show, situations may vary widely and are shaped by psychosocial factors, biographical and cultural roots, spiritual beliefs, personalities, and living conditions. It would not meet the needs of the patients and their families to generalize a view that WTDs, if they are founded on SPB, can (or even must) be resolved by care and support. Sometimes, the patient has important personal reasons for rejecting care.

The main objective of clinical interaction in advanced palliative care situations is to foster the best possible quality of life. Facing the coming end of life, it can make sense, or even be necessary, from the patient's existential perspective to allow room for there to be a wish to die. In a spiritual dimension, it may be closely connected with acceptance,23Ohnsorge, K., Rehmann‐Sutter, C., Streeck, N., Widdershoven, G., & Gudat, H. (2017). Was bedeutet es, das eigene Sterben zu `akzeptieren'? Ergebnisse aus einer qualitativen Studie mit 62 Palliativpatientinnen und ‐patienten. Zeitschrift für Palliativmedizin, 18, 144‐151. doi: https://doi.org/10.1055/s-0043-100549. and can be a relief. Patients and families should be reassured that having a WTD due to feeling one is a burden is nothing abnormal and, above all, it is not morally wrong.

Clinicians may ask whether a wish to hasten death is undermined by being based primarily on an SPB. In such cases is it always acceptable as a compelling reason for a form of assisted dying (such as stopping life‐sustaining treatments)? From an ethical point of view that focuses on the importance of care and relationship,24Rehmann‐Sutter, C. (2016) Wünsche am Lebensende wahrnehmen—Ethische Impulse palliativer Versorgung. In Conradi, E., & Vosman, F. (eds) *Praxis der Achtsamkeit. Schlüsselbegriffe der Care‐Ethik* (pp. 167–187) Frankfurt am Main: Campus. the first step in a process of clarification would be to reassure patients that they are welcomed and respected as equal members of our society and that people around them (including the medical system) are willing to handle this difficult life situation together with them.

But how to respond if the patient's self‐perception is so strained that she feels overwhelmed by the situation, including its social roles and demands? And if she is tired of enduring it? Advances in medicine have led to such situations— which are also very hard for informal and professional caregivers. How the situation can be resolved *or endured* together needs to be considered carefully in each case. This may also imply that some patients wish to put their wish to end life into some kind of practice.

On the other hand, some wishes in life cannot be fulfilled, even in critical situations, and vulnerable patients (and family systems) need to be protected. It could, for example, be questioned whether patients with organ failure belong to a more vulnerable group than those with other diseases. They seem to struggle with SPB (as they did in our study), but are conditioned and encouraged by professionals to fight. Seeing themselves rather distant from the end of life, these patients probably cannot allow attitudes or wishes about the end of life to emerge. Becoming aware of the advanced palliative situation too late may then cause an unnecessary existential crisis, heavily burdening all partners, and generating WTD that produce conflicts, distress, and prolonged grief for those who have cared and will remain.

Thus, those involved should explore how the patient and the family can be unburdened as far as possible. This process needs time, creativity, comprehension, and trusting relationships. It underlines the importance of early palliative care adapted to the needs of the patient–caregiver unit, and of thoroughly integrating narrative approaches into medical practice.

